# Intestinal Development and Histomorphometry of Broiler Chickens Fed *Trichoderma reesei* Degraded Date Seed Diets

**DOI:** 10.3389/fvets.2020.00349

**Published:** 2020-08-18

**Authors:** Salem R. Alyileili, Khaled A. El-Tarabily, Ibrahim E. H. Belal, Wissam H. Ibrahim, Mohsin Sulaiman, Ahmed S. Hussein

**Affiliations:** ^1^Department of Integrative Agriculture, College of Food and Agriculture, United Arab Emirates University, Al Ain, United Arab Emirates; ^2^Department of Biology, College of Science, United Arab Emirates University, Al Ain, United Arab Emirates; ^3^Khalifa Center for Genetic Engineering and Biotechnology, United Arab Emirates University, Al Ain, United Arab Emirates; ^4^College of Science, Health, Engineering and Education, Murdoch University, Murdoch, WA, Australia; ^5^Department of Food, Nutrition and Health, College of Food and Agriculture, United Arab Emirates University, Al Ain, United Arab Emirates

**Keywords:** broilers, degraded date pits, intestine development, pancreatic enzymes, villus

## Abstract

A study was conducted to investigate the impact of degraded date pits (DDP) on the development and morphology of the intestine in broilers. *Trichoderma reesei* was used to produce the DDP using a solid-state degradation method. One hundred and eighty broilers were divided into six treatments in triplicate groups of 10 chicks each. The dietary treatments were: positive control with corn-soy basal diet, negative control with corn-soy basal diet + 20% oxytetracycline at 0.05%, corn-soy basal diet + 10% DDP, corn-soy basal diet + 0.2% mannan-oligosaccharides (MOS), corn-soy basal diet + 0.2% mannose and corn-soy basal diet + 0.1% mannose for 6 weeks. The results indicate that a 10% DDP diet increased the activities of the pancreatic enzymes, the villus length, and the villus/crypt ratio, and decreased the crypt depth of the intestine. In conclusion, when compared to oxytetracycline and MOS, DDP can be used as a replacement for antibiotic growth promoters for broilers while improving gut development and intestinal health.

## Introduction

In poultry, the gut is a critical site where nutrient uptake takes place. In recent years, intense studies have been focused on gut health for increasing poultry production (1). Hindered functioning of the gut causes impaired digestion, nutrient malabsorption, and decreased growth performance of poultry. The subtherapeutic use of antibiotics increases the thickening of the intestine, which promotes nutrient absorption and reduces the beneficial bacterial count (2). Therefore, recent research has evaluated the use of feed supplements to decrease the adverse effects of prolonged use of antibiotics for improving growth performance and intestine development (3). Considerable interest has been shown in the use of alternatives such as probiotics, prebiotics, synbiotics, herbal medicines, and fermented feeds to improve broiler performance and to influence the bacterial ecology of their gastrointestinal tract.

Prebiotics may be included as a substitute for antibiotics in order to limit the negative impact of antibiotics on animal and human health, and the environment. Prebiotics are non-digestible food ingredients and widely used in animal nutrition (4). Prebiotics produced naturally from plant waste may be useful and affordable sources for animal nutrition to improve the gut ecosystem and thus animal performance. Prebiotics positively affect the host by influencing the growth of intestinal bacteria and thereby stimulating immunity and overall performance (5). A commercial prebiotic yeast extract ingredient, namely mannan-oligosaccharides (MOS) has been widely used in poultry rations, which regulates intestinal development and prevents the growth of pathogenic microbes in chicken gastrointestinal (GI) tracts (6). Enhanced immune system and intestinal mucosa with improved morphology and growth were observed in chickens fed MOS included diets. In domesticated animals, prebiotics in the form of non-digestible inulin-type fructans are widely used. The beneficial microbial populations in birds' gut were improved by the supplementation of prebiotics or fermentable sugars instead of antibiotics, and nowadays the use of prebiotics seems to be popular in the poultry industry (7).

Date palm (*Phoenix dactylifera* L.) is one of the major fruit crops in most Arabian countries. Date pits (DP) are widely produced as byproducts from date confectionery industry. In poultry feed, the imported corn or other cereals could be partly replaced with DP. About 11–18% of date fruit weight is the seed which is composed of carbohydrates, dietary fiber, fat, ash, and protein (8). The growth performance of broilers fed DP diet at levels ranging from 5 to 27% suggested no deleterious effect (9). The body weight gain (BWG) and feed conversion ratio (FCR) of chicks fed a diet containing 10–15% DP were significantly increased after the first 2 weeks of the experiment (8, 10). Several reports showed the beneficiary effect of date fiber on growth performance and the carcass characteristics of broilers and layers (11–14).

Solid-state degradation (SSD) of DP with exogenous microbial enzymes such as xylanases enhances the production of simpler forms of carbohydrate molecules from fibers present in the DP (15). A considerable investigation is currently concentrated on the cellulolytic filamentous fungus, *Trichoderma reesei*. Lignocellulose and galactomannan found in DP act as the natural substrates of *T. reesei* which enhances growth of microbes and activation of several catabolic enzymes. When DP is subjected to degradation, enzymes, such as cellulases, hemicellulases, and pectinases, secreted by *T. reesei* catalyzes the degeneration of complex substrates to simple sugars, thus enhancing its nutritional composition. Degradation improves the chemical constituents of DP and SSD is one of the reasonable methods to prepare degraded date pits (DDP) by using *T. reesei*. Microbially degraded feeds and enzymes can be better utilized by animals and improve the growth performance and bacterial ecology of their gastrointestinal tract (16). The advantage of using enzymatically degraded prebiotics such as DDP in animal feed is that it promotes the beneficial role of non-digestible sugars in increasing the immune system of the animal.

DDP can act as a prebiotic like MOS which modulates the ecosystem of the animal gut. The mechanism for regulating the bacterial population in the intestine is by binding it to pathogenic bacteria, which possess type-1 fimbriae, such as *Escherichia coli* and *Salmonella* species, and by blocking bacterial lectin. It also regulates the production of cytokines and antibodies, improving gut development and overall broiler health. The prebiotic molecules will be degraded into individual sugars and byproducts after absorption and then eliminated from the animal's body through the metabolic process (17). This property of a natural growth promoter derived from DP is a milestone in animal feed research. Hence, the objective of the study was to investigate the impact of DDP on carcass characteristics, intestinal development, morphology, and pancreatic digestive enzyme activity in broilers and to compare its effect with MOS.

## Materials and Methods

### Chicks, Diets and Experimental Design

Chicks were managed and handled according to animal welfare guidelines of the United Arab Emirates (UAE) University Research Ethics Committee, which recommends the rights and welfare of the experimental animals.

Fresh DP (*Phoenix dactylifera* L.), Khalas variety were obtained from Al Saad date processing factory in Al-Ain, UAE. Each seed was about 2–2.5 cm long and 6–8 mm thick. A medium-size mill (Skiold A/S, Kjeldgaardsvej 3, Saeby 9300, Denmark) was used to grind the DP. The size of the pits was reduced to about 1 mm.

The *T. reesei* used in the current study was cultivated as described (18). Four lyophilized *T. reesei* ampoules were purchased from Deutsche Sammlung von Mikroorganismen und Zellkulturen GmbH (DSMZ), Braunschweig, Germany. A subsample from the re-hydrated *T. reesei* culture was transferred to potato dextrose broth (PDB; Lab M Ltd., Lancashire, United Kingdom) amended with 250 μg mL^−1^ chloramphenicol (Sigma–Aldrich Chemie GmbH, Taufkirchen, Germany) and 100 μg mL^−1^ streptomycin sulfate (Sigma–Aldrich). A rotary shaker (Model G76, New Brunswick Scientific, Edison, NJ, USA) was used to incubate the flasks at 250 rpm at 25 ± 2°C in the dark for 7 days. The flasks were visually examined daily for fungal growth.

Pure *T. reesei* cultures were maintained on potato dextrose agar (PDA; Lab M Ltd.) plates. The incubation of the plates was carried out in the dark at 25 ± 2°C for 7 days and visually examined daily for fungal growth. The fungus was maintained on full spelling for PDA plates and stored at 4°C.

The *T. reesei* DDP was produced using a SSD method inside an incubator, as described (18). Briefly, the ground DP were mixed, cleaned, and sterilized at 121°C for 30 min. Sterilized DP were added to each cone of the SSD system. The starter culture of *T. reesei* prepared on PDA was added to each cone of the SSD system containing the sterilized DP. More cultures and sterilized DP were added in layers to the SSD system until the solution volume reached 8 L per cone. A continuous supply of disinfected moistened air to the SSD system was from a small air pump and a Aquafine ultraviolet lamp. The SSD system was kept in the dark with 90% humidity at 30 ± 2°C for 3 weeks. At the end of the third week, the procedure of degradation was stopped, and the DDP collected and kept in a refrigerator (4°C) until use. In our previous study, we analyzed the chemical composition of DDP and published the results (19).

Diets were formulated as indicated in Tables 1, 2. All diets were isonitrogenous and isocaloric. The calculated nutrient composition of the dietary treatments was based on the ingredient composition tables (20). All feed ingredients were ground to a suitable size and mixed in a commercial mixer (Hobart mixer, HL1400, USA) for 20 min. Vitamin and mineral premixes, fish meal, and oil were added gradually with continuous mixing. The wet mix was then passed through a commercial mixer for 15 min for homogenous distribution of the nutrients and particle sizes. The wet mix was then used for diet preparation.

**Table 1:** Composition and calculated chemical analysis of experimental starter diets on dry matter basis for broilers.

**Ingredients,%**	**T1**	**T2**	**T3**	**T4**	**T5**	**T6**
Yellow corn	55.4	55.35	44.6	55.2	55.3	55.2
Soybean meal	36	36	33.25	36	36	36
Salt	0.4	0.4	0.38	0.4	0.4	0.4
Limestone	1.1	1.1	1.1	1.1	1.1	1.1
Di-calcium phosphate	1.56	1.56	1.2	1.56	1.56	1.56
Vit. + Min. Premix^a^	1	1	1	1	1	1
DL-methionine	0.24	0.24	0.25	0.24	0.24	0.24
Lysine	0	0	0.1	0	0	0
Corn oil	2	2	5.02	2	2	2
Fish meal	2.3	2.3	3.1	2.3	2.3	2.3
Oxytetracycline	0	0.05	0	0	0	0
Degraded date pits	0	0	10	0	0	0
Mannan-oligosaccharides	0	0	0	0.2	0	0
Mannose	0	0	0	0	0.2	0.1
**Calculated and determined chemical composition (% of DM basis)**						
ME, MJ/kg diet	12.82	12.82	12.95	12.90	12.74	12.69
Crude protein	21.33	21.33	21.2	21.30	21.12	21.30
Methionine	0.46	0.46	0.45	0.45	0.44	0.45
Methionine + cysteine	0.85	0.85	0.95	0.97	0.87	0.89
Lysine	1.07	1.07	1.33	1.29	1.17	1.15
Threonine	0.85	0.85	0.87	0.88	0.86	0.86
Crude fiber	3.36	3.36	4.19	4.36	3.0	3.36
Calcium	0.89	0.89	0.92	0.90	0.92	0.94
Available phosphorus	0.46	0.46	0.45	0.4	0.51	0.55
Sodium	0.21	0.21	0.22	0.20	0.21	0.21

*T1, Positive control; T2, Negative control; T3, CSBD + 10% DDP; T4, CSBD + 0.2% MOS; T5, CSBD + 0.2% mannose; T6, CSBD + 0.1% mannose*.

a *Supplementary levels of vitamins and trace elements (per Kg):Vitamin A, 5,484 IU; Vitamin D_3_, 2,822 IU; Vitamin E (as DL alpha-tocopherol acetate) 26 IU; Vitamin K (as menadione sodiumbisulfite), 4.38 mg; Thiamine, 5.94 mg; Riboflavin, 6.2 mg; Pyridoxine, 4.5 mg; Cyanocobalamin, 0.14 mg; Niacin (as nicotinic acid), 44.1 mg; D-pantothenic acid, 15 mg; Folic acid, 990 μg, Biotin, 0.23 mg; Iron, 120 mg; Copper, 8 mg; Manganese, 83 mg; Cobalt, 5 mg; Zinc, 60 mg; Iodine, 1.11 mg; Selenium, 300 μg. CSBD, corn-soy basal diet*.

**Table 2:** Composition and calculated chemical analysis of experimental finisher diets on dry matter basis for broilers.

**Ingredients,%**	**T1**	**T2**	**T3**	**T4**	**T5**	**T6**
Yellow corn	64.6	64.55	52.14	64.4	64.4	64.5
Soybean meal	28.4	28.4	26.0	28.4	28.4	28.4
Salt	0.42	0.42	0.33	0.42	0.42	0.42
Limestone	1.33	1.33	1.15	1.33	1.33	1.33
Di-calcium phosphate	1.05	1.05	0.8	1.05	1.05	1.05
Vit. + Min. Premix^a^	0.2	0.2	0.2	0.2	0.2	0.2
DL-methionine	0.2	0.2	0.3	0.2	0.2	0.2
Lysine	0.1	0.1	0.18	0.1	0.1	0.1
Corn oil	2.5	2.5	5.9	2.5	2.5	2.5
Fish meal	1.2	1.2	3	1.2	1.2	1.2
Degraded date pits	0	0	10	0	0	0
Oxytetracycline	0	0.05	0	0	0	0
Mannan-oligosaccharides	0	0	0	0.2	0	0
Mannose	0	0	0	0	0.2	0.1
Total	100	100	100	100	100	100
**Calculated and determined chemical composition (% of DM basis)**						
ME, MJ/kg diet	13.12	13.12	13.12	13.12	13.12	13.12
Crude protein	17.75	17.55	17.72	17.75	17.75	17.75
Methionine	0.47	0.47	0.49	0.47	0.47	0.47
Methionine + cysteine	0.86	0.86	0.84	0.86	0.86	0.86
Lysine	1.05	1.05	1.1	1.05	1.05	1.05
Threonine	0.71	0.71	0.73	0.71	0.71	0.71
Crude fiber	3.09	3.09	3.92	3.09	3.09	3.09
Calcium	0.82	0.82	0.80	0.82	0.82	0.82
Available phosphorus	0.39	0.39	0.38	0.39	0.39	0.39
Sodium	0.20	0.20	0.20	0.20	0.20	0.20

*T1, Positive control; T2, Negative control; T3, CSBD + 10% DDP; T4, CSBD + 0.2% MOS; T5, CSBD + 0.2% mannose; T6, CSBD + 0.1% mannose*.

a *Supplementary levels of vitamins and trace elements (per Kg):Vitamin A, 5,484 IU; Vitamin D_3_, 2,822 IU; Vitamin E (as DL alpha-tocopherol acetate) 26 IU; Vitamin K (as menadione sodiumbisulfite), 4.38 mg; Thiamine, 5.94 mg; Riboflavin, 6.2 mg; Pyridoxine, 4.5 mg; Cyanocobalamin, 0.14 mg; Niacin (as nicotinic acid), 44.1 mg; D-pantothenic acid, 15 mg; Folic acid, 990 μg, Biotin, 0.23 mg; Iron, 120 mg; Copper, 8 mg; Manganese, 83 mg; Cobalt, 5 mg; Zinc, 60 mg; Iodine, 1.11 mg; Selenium, 300 μg. CSBD, corn-soy basal diet*.

### Digestive Enzyme Activity in Pancreas, and Weight, Length, and Histomorphology of the Intestinal Segments

A total of 180 Brazilian “Cobb 500” broiler chicks were divided into six treatments, each containing three replicates of 10 unsexed broilers. Birds within each treatment were designated to three replicate groups (*n* = 10/group). Chickens were housed as 10 chicks per cage (50 cm × 45 cm × 45 cm) in an environmentally controlled house. Feed and water were given on an *ad libitum* basis. The light-dark cycle was 23:1 daily from the 4d of the experiment. Vaccination and medical care were carried out under the supervision of veterinaries. The experiment lasted for 42 days.

The treatments consisted of positive control diet (T1), negative control diet (T2), corn-soy basal diet (CSBD) + 10% DDP (T3), CSBD + 0.2% MOS (T4), CSBD + 0.2% mannose (T5), and CSBD + 0.1% mannose (T6). The pancreatic and gut samples were collected from two broilers of each replicate per treatment (6 birds/treatment) and were slaughtered randomly at both 21 and 42 days of age. Samples of the pancreas and different intestinal segments (duodenum, jejunum, and ileum) were collected at different periods. The amylase, protease, and lipase activities were measured using the method of Gertler and Nitsan (21). The weight (g) and length (cm) of intestinal segments were calculated by the method of Ling et al. (22). The histomorphology of broiler intestine was analyzed by the method of Sun et al. (23).

### Statistical Analysis

Data of each experiment were subjected to the analysis of variance (one-way ANOVA) using a general linear model (GLM), and the mean comparisons were performed using Duncan's multiple range test to compare significant differences between the means for all analyses. Analysis of variance was completed using the software SPSS for Windows (Version 20.0, SPSS Inc., Chicago, IL, USA). The replicate was the experiment unit. Data in percentage were transformed to arcsine before analysis.

## Results

### Effect of Different Dietary Treatments on Carcass Characteristics and Organ Weight of Broilers

Table 3 shows the effect of different dietary treatments on the organ weight of broilers. The results showed that there was no significant difference between the live weights of broilers fed different diets. The cold carcass weight and dressing percentage were similar in all dietary treatment groups. The weight of the heart, liver, breast, and thighs was significantly raised in all dietary treatments compared to broilers fed control diets.

**Table 3:** Effect of different dietary treatments on carcass characteristics and organ weight of broilers on day 42 of age (*n* = 6 chicks per treatment, mean ± SE).

**Parameters**	**Dietary treatments**					
	**T1**	**T2**	**T3**	**T4**	**T5**	**T6**
Live weight, g	2,279 ± 8.0^a^	2,371 ± 22^a^	2,368 ± 12^a^	2,340 ± 14^a^	2,302.6 ± 83^a^	2,261 ± 14^a^
Carcass, g	1,550 ± 24.6^a^	1,604 ± 12^a^	1,696 ± 12^a^	1,665 ± 10^a^	1,628 ± 59^a^	1,561 ± 39^a^
Dressing, %	68.0 ± 1.3^a^	67.7 ± 1.5^a^	71.7 ± 0.18^a^	71.0 ± 0.7^a^	70.7 ± 0.6^a^	69.0 ± 1.4^a^
Breast, g	706 ± 7.6^c^	760 ± 7.6^ab^	762 ± 9.2^a^	755 ± 5^ab^	722 ± 7^bc^	704 ± 10.8^c^
Thigh, g	712 ± 14^bc^	772 ± 4.0^a^	776 ± 7.8^a^	755 ± 5.7^ab^	735 ± 7.6^abc^	711 ± 11^c^
Wings, g	182 ± 3.7^a^	190 ± 2.6^a^	190 ± 2.0^a^	190 ± 3.0^a^	182 ± 2.0^a^	179 ± 3.5^a^
Liver, g	49.3 ± 0.3^c^	55.3 ± 0.4^a^	55.5 ± 0.7^a^	54.6 ± 0.4^a^	48.7 ± 0.3^c^	49.3 ± 1.5^c^
Heart, g	14.2 ± 0.3^c^	15.3 ± 0.18^a^	14.8 ± 0.06^b^	14.6 ± 0.03^b^	14.7 ± 0.07^b^	13.8 ± 0.3^c^
Gizzard, g	30.9 ± 2.16^a^	32.2 ± 3.23^a^	33.5 ± 3.33^a^	34.0 ± 2.97^a^	30.0 ± 4.65^a^	30.4 ± 2.92^a^
Spleen, g	2.41 ± 0.09^a^	2.82 ± 0.2^a^	2.91 ± 0.3^a^	3.10 ± 0.2^a^	2.70 ± 0.4^a^	2.51 ± 0.1^a^
Proventriculus, g	4.29 ± 0.46^a^	5.75 ± 0.53^a^	6.21 ± 0.38^a^	6.41 ± 0.48^a^	5.40 ± 0.77^a^	6.10 ± 0.63^a^

*Within rows, values followed by the same letter are not significantly (P>0.05) different according to Duncan's multiple range test; T1, Positive control; T2, Negative control; T3, CSBD + 10% DDP; T4, CSBD + 0.2% MOS; T5, CSBD + 0.2% mannose; T6, CSBD + 0.1% mannose. CSBD, corn-soy basal diet; DDP, degraded date pits; MOS, mannan-oligosaccharides*.

### Effect of Different Dietary Treatments on the Weight of Duodenum, Jejunum and Ileum

The weight of duodenum at 21 and 42 days of age was increased significantly in only the 10% DDP diet-fed broilers and the 0.2% MOS diet-fed broilers when compared to other groups at the same age (Table 4). On day 21 of the experiment, the weight of jejunum was similar in all diet-fed broilers, while on day 42 the weight of jejunum was increased significantly in 10% DDP diet-fed broilers and 0.2% MOS diet-fed broilers when compared to the positive control. In negative control broilers and mannose diet-fed broilers, the weight of jejunum was decreased, and the result was comparable to positive control diet-fed broilers. The weight of the ileum at 21 and 42 days was significantly higher in 10% DDP diet-fed broilers and 0.2% MOS diet-fed broilers when compared to other treatments (Table 4).

**Table 4:** Effect of different dietary treatments on weight (g) and length (cm) of duodenum, jejunum and ileum of broilers (*n* = 6 chicks per treatment, mean ± SE).

**Parameters**	**Dietary treatments**					
	**T1**	**T2**	**T3**	**T4**	**T5**	**T6**
**Weight on day 21**						
Duodenum, g	9.78 ± 0.55^b^	10.47 ± 0.61^b^	11.96 ± 0.70^a^	12.04 ± 0.35^a^	9.73 ± 0.43^b^	9.28 ± 0.34^b^
Jejunum, g	17.3 ± 1.00^a^	16.2 ± 0.77^a^	19.7 ± 1.29^a^	18.6 ± 0.77^a^	19.3 ± 0.47^a^	18.2 ± 0.71^a^
Ileum, g	12.4 ± 0.71^b^	13.3 ± 0.75^b^	15.7 ± 0.61^a^	15.2 ± 0.40^a^	12.9 ± 0.19^b^	11.9 ± 5.81^b^
**Weight on day 42**						
Duodenum, g	3.78 ± 0.20^b^	4.39 ± 0.28^ab^	5.32 ± 0.12^a^	6.23 ± 0.42^a^	4.89 ± 0.08^ab^	5.01 ± 0.06^ab^
Jejunum, g	8.38 ± 0.45^b^	10.94 ± 0.75^b^	14.89 ± 0.45^a^	16.45 ± 0.58^a^	10.84 ± 0.21^b^	10.12 ± 0.55^b^
Ileum, g	3.68 ± 0.09^b^	5.71 ± 0.45^ab^	8.83 ± 0.39^a^	8.13 ± 0.17^a^	5.81 ± 0.36^ab^	6.27 ± 0.49^ab^
**Length on day 21**						
Duodenum, cm	30.9 ± 1.30^b^	34.3 ± 1.79^b^	42.2 ± 1.02^a^	47.5 ± 1.20^a^	30.6 ± 0.66^b^	30.4 ± 0.50^b^
Jejunum, cm	74.0 ± 1.88^b^	70.5 ± 2.34^b^	90.9 ± 3.32^a^	94.3 ± 4.94^a^	78.1 ± 3.77^b^	76.7 ± 3.35^b^
Ileum, cm	64.0 ± 2.40^b^	64.8 ± 21.46^b^	71.8 ± 2.86^a^	69.1 ± 2.08^a^	68.0 ± 0.19^b^	66.9 ± 2.26^b^
**Length on day 42**						
Duodenum, cm	8.42 ± 0.47^b^	7.34 ± 0.41^b^	10.04 ± 0.68^a^	11.82 ± 0.17^a^	8.75 ± 0.53^b^	9.62 ± 0.13^b^
Jejunum, cm	28.0 ± 0.97^b^	24.5 ± 1.71^b^	36.5 ± 2.70^ab^	45.7 ± 2.78^a^	26.9 ± 1.70^b^	30.0 ± 1.30^b^
Ileum, cm	23.9 ± 0.85^b^	21.5 ± 0.72^b^	33.6 ± 1.78^a^	32.8 ± 2.08^a^	26.0 ± 0.61^b^	23.6 ± 0.76^b^

*Within rows, values followed by the same letter are not significantly (P>0.05) different according to Duncan's multiple range test; T1, Positive control; T2, Negative control; T3, CSBD + 10% DDP; T4, CSBD + 0.2% MOS; T5, CSBD + 0.2% mannose; T6, CSBD + 0.1% mannose. CSBD, corn-soy basal diet; DDP, degraded date pits; MOS, mannan-oligosaccharides*.

### Effect of Different Dietary Treatments on Length of Duodenum, Jejunum, and Ileum

The length of the duodenum was increased significantly in 10% DDP diet-fed broilers and 0.2% MOS diet-fed broilers when compared to the positive control (Table 5). On day 21 and day 42, in broilers fed a CSBD with or without antibiotic and a mannose diet, the length of the duodenum was smaller when compared to 10% DDP diet-fed broilers and MOS diet-fed broilers. The result was comparable to the positive control. On day 21, the length of jejunum was increased significantly in 10% DDP diet-fed broilers and 0.2% MOS diet-fed broilers when compared to other treatments. The other treatments were substantially similar. On day 42 of the experiment, the jejunum length was increased significantly in only MOS diet-fed broilers when compared to other treatments except for those fed a 10% DDP diet. The 10% DDP treatment was intermediate. On day 21 of the experiment, the length of ileum was the same in all treatments. On day 42 of the trial, the length of ileum was shown to be increased significantly in broilers fed a 10% DDP diet and a 0.2% MOS diet when compared to other treatments.

**Table 5:** Effect of different dietary treatments on histomorphometry of duodenum, jejunum and ileum of broilers (*n* = 6 chicks per treatment, mean ± SE).

**Parameters**	**Dietary treatments**					
	**T1**	**T2**	**T3**	**T4**	**T5**	**T6**
**Morphology of duodenum on day 21**						
Villus height, μm	929 ± 13.32^b^	884 ± 13.32^b^	1,023 ± 19.37^a^	1,001 ± 16.58^a^	970 ± 18.21^ab^	10,161 ± 15.28^ab^
Crypt depth, μm	125 ± 1.91^a^	138 ± 3.83^a^	103 ± 1.21^b^	109 ± 1.98^b^	117 ± 3.00^ab^	106 ± 1.70^b^
Villus/crypt ratio, μm/μm	7.79 ± 1.13^b^	7.04 ± 1.51^b^	10.75 ± 2.10^a^	10.30 ± 1.80^a^	8.92 ± 1.59^b^	9.84 ± 1.70^b^
**Morphology of duodenum on day 42**						
Villus height, μm	1,054 ± 15.75^a^	1,038 ± 18.21^a^	1,155 ± 27.70^a^	1,130 ± 24.76^a^	1,147 ± 27.16^a^	1,163 ± 29.50^a^
Crypt depth, μm	119 ± 5.76^a^	127 ± 9.21^a^	116 ± 4.97^a^	105 ± 4.50^a^	113 ± 7.91^a^	123 ± 4.86^a^
Villus/crypt ratio, μm/μm	9.52 ± 1.29^a^	8.75 ± 0.97^a^	10.81 ± 1.92^a^	11.64 ± 2.00^a^	11.00 ± 1.57^a^	10.20 ± 1.89^a^
**Morphology of jejunum on day 21**						
Villus height, μm	715 ± 29.67^b^	685 ± 25.58^b^	941 ± 34.91^a^	877 ± 32.93^a^	815 ± 29.91^ab^	907 ± 27.40^a^
Crypt depth, μm	111 ± 14.08^a^	111 ± 7.92^a^	132 ± 9.04^a^	123 ± 10.16^a^	108.9 ± 5.48^a^	128 ± 7.58^a^
Villus/crypt ratio, μm/μm	6.74 ± 0.31^a^	6.87 ± 1.51^a^	7.50 ± 0.75^a^	7.58 ± 0.88^a^	8.21 ± 1.79^a^	7.86 ± 1.72^a^
**Morphology of jejunum on day 42**						
Villus height, μm	886 ± 24.62^b^	863 ± 27.21^b^	1,143 ± 33.76^a^	1,138 ± 24.77^a^	1,117 ± 20.16^a^	1,149 ± 29.50^a^
Crypt depth, μm	122 ± 4.56^a^	139 ± 6.36^a^	93.1 ± 2.50^b^	96.3 ± 4.50^b^	119 ± 5.53^a^	94.6 ± 3.46^b^
Villus/crypt ratio, μm/μm	7.44 ± 0. 45^b^	6.38 ± 0.34^b^	12.85 ± 1.51^a^	12.72 ± 2.00^a^	9.76 ± 0.71^ab^	12.82 ± 1.61^a^
**Morphology of ileum on day 21**						
Villus height, μm	514 ± 13.80^b^	492 ± 9.23^b^	582 ± 10.39^a^	565 ± 8.69^a^	516 ± 6.50^ab^	562 ± 11.38^a^
Crypt depth, μm	89.0 ± 4.11^a^	101.4 ± 5.26^c^	60.4 ± 2.67^b^	64.0 ± 1.90^b^	74.2 ± 2.49^ab^	70.1 ± 1.77^ab^
Villus/crypt ratio, μm/μm	5.98 ± 0.44^b^	5.04 ± 0.33^b^	10.10 ± 0.93^a^	9.01 ± 0.72^a^	7.62 ± 1.54^ab^	8.31 ± 0.74^ab^
**Morphology of ileum on day 42**						
Villus height, μm	764 ± 21.52^b^	733 ± 15.48^b^	816 ± 17.64^a^	821 ± 19.25^a^	820 ± 13.26^a^	841 ± 19.25^a^
Crypt depth, μm	136 ± 7.91^a^	150 ± 8.57^a^	98.5 ± 3.67^b^	92.3 ± 2.60^b^	105.6 ± 4.50^ab^	114.4 ± 5.42^ab^
Villus/crypt ratio, μm/μm	5.94 ± 0.60^b^	5.11 ± 0.34^b^	8.53 ± 0.50^a^	9.06 ± 0.42^a^	8.27 ± 0.91^ab^	7.54 ± 0.32^ab^

*Within rows, values followed by the same letter are not significantly (P>0.05) different according to Duncan's multiple range test; T1, Positive control; T2, Negative control; T3, CSBD + 10% DDP; T4, CSBD + 0.2% MOS; T5, CSBD + 0.2% mannose; T6, CSBD + 0.1% mannose. CSBD, corn-soy basal diet; DDP, degraded date pits; MOS, mannan-oligosaccharides*.

### Effect of Different Dietary Treatments on Histomorphology of Intestine

Results showed that the villus height, crypt depth and villus crypt ratio of the duodenum were significantly raised in broilers fed a 10% DDP diet and 0.2% MOS diet on day 21 when compared to control-diet broilers and mannose diet-fed broilers. On day 42, there was no significant variation between villus height, crypt depth, and villus crypt ratio of the duodenum of broilers fed different dietary treatments (Table 5). On day 21 and day 42, the villus height of jejunum was significantly increased in broilers fed 10% DDP diet compared to other treatments. The crypt depth of jejunum was decreased significantly in broilers fed 10% DDP diet on day 42, and no significant change was observed in the villus crypt ratio of different dietary treatments during the experimental period (Table 5). In the ileum, the villus height was significantly increased in 10% DDP diet-fed broilers, 0.2% MOS diet-fed broilers, and 0.1% mannose diet-fed broilers on days 21 and 42. During the experimental period, crypt depth of ileum was decreased in broilers fed 10% DDP diet and a mannose diet-fed broilers. In broilers fed a 10% DDP diet and a 0.2% MOS diet the villus/crypt ratio of ileum was significantly increased on days 21 and 42 when compared to the positive and negative control (Table 5).

### Effect of Different Dietary Treatments on Pancreatic Enzymes

The activity of the pancreatic enzymes amylase and lipase were observed to be significantly higher in 10% DDP diet-fed broilers and 0.2% MOS diet-fed broilers when compared to other treatments. The activity of protease was similar in all treatments (Table 6).

**Table 6:** Effect of different dietary treatments on the activity of digestive enzymes (U mg^−1^ protein) of the pancreas of broiler chickens (*n* = 6 chicks per treatment, mean ± SE).

	**Dietary treatments**					
**Parameters**	**T1**	**T2**	**T3**	**T4**	**T5**	**T6**
Amylase	411 ± 20.70^b^	470 ± 16.54^b^	526 ± 19.94^a^	517 ± 17.49^a^	496 ± 16.58^b^	502 ± 15.27^b^
Protease	4,926 ± 262.70^a^	5,054 ± 108.28^a^	5,577 ± 139.27^a^	5,305 ± 164.69^a^	5,101 ± 118.24^a^	5,166 ± 124.61^a^
Lipase	58.4 ± 5.21^b^	52.3 ± 7.74^b^	83.2 ± 12.49^a^	96.2 ± 12.44^a^	60.5 ± 3.73^b^	59.8 ± 4.80^b^

*Within rows, values followed by the same letter are not significantly (P>0.05) different according to Duncan's multiple range test; T1, Positive control; T2, Negative control; T3, CSBD + 10% DDP; T4, CSBD + 0.2% MOS; T5, CSBD + 0.2% mannose; T6, CSBD + 0.1% mannose. CSBD, corn-soy basal diet; DDP, degraded date pits; MOS, mannan-oligosaccharides*.

## Discussion

The present results indicated that microbial treatment leads to the catabolism and degradation of main macronutrients such as carbohydrates, protein, and fatty acids, accompanied by an increase in simple sugars, free amino acids, and organic acids. The crude fiber content of DDP was found to be 20.8%, ash (2.09%), crude fat (7.2%), protein (5.56%), and total carbohydrate (87.2%). Neutral detergent fiber (NDF) and acid detergent fiber (ADF) were found to be 74.6 and 45.7%, respectively. Monosaccharide composition of fiber showed that the degradation with *T. reesei* significantly (*P* < 0.05) enhanced the glucose and mannose content of cellulose, hemicellulose, and lignin. Pectin, total carbohydrate, and the MOS content were also increased in DDP, in which galactose and mannose were the major neutral sugars. Among the studied minerals, potassium, calcium, magnesium, sulfur, and phosphorus were predominant. The phenolic and flavonoid contents of DDP significantly (*P* < 0.05) increased after treatment with *T. reesei*. Among the studied minerals in DDP, potassium, calcium, magnesium, sulfur, and phosphorus were predominant. DDP also showed marked activities of antioxidant such as 2,2′-azino-bis (3-ethylbenzothiazoline 6 sulphonic acid) (ABTS), Ferric Reducing Antioxidant Power (FRAP) assays, and 2,2-diphenyl-1-picrylhydrazyl (DPPH) (19).

Several studies have shown the beneficial influences of prebiotics on carcass characteristics of broilers. In the present study, the dressing yield for broiler fed 10% DDP was 71.6%, and 71.0% for those fed 0.2% MOS. Increases in carcass output of broilers of our study were higher than those reported by Rao et al. (24), who concluded that the dressing yield ranged from 63.7 to 66.7% of commercial Cobb broilers. The carcass yield was 71.0% at 42 days of age, which was similar to the value observed by Abdel-Raheem and Abd-Allah (25). They also reported that broiler thigh and breast weight were elevated after adding single or combined dietary supplements of MOS and probiotics. Yeast based MOS supplementation improved carcass characteristics and blood biochemical parameters in broiler chickens (26). Similar results were seen in our present study. The current findings agreed with (27–29), who reported that prebiotics, probiotics, and synbiotics had no significant positive effect on carcass yields of quails and broilers. MOS supplementation in broiler diets had no significant effect on dressing percentage and gizzard, spleen, proventriculus, and bursa weight (30). The addition of MOS at a dose of 3 g/kg feed improved the carcass characteristics and intestinal microbial ecology of growing Japanese quails (25). Broilers fed palm kernel meal diet reduces the relative size of broiler organs, which reduces the relative weight of such organs (31).

The development of intestine is a critical factor, and the weight of intestinal parts reflects the physiological status and function of broilers (32). Intestine enhances immunization and regulates digestion and absorption of nutrients. The development of the intestine was reflected by the relative length and weight of organs. In our study, the increased length and weight of the intestine proves that dietary supplementation with 10% DDP stimulates the intestinal development of broilers. It is established that the longer the length of the intestine the greater will be the absorption of nutrients. The role of intestinal parts, mainly the jejunum in digestion and absorption of broilers, is reflected by an increase in length and height of its epithelial cells (33). Broilers fed DDP had longer jejunum, which showed supplementation of broiler diet with 10% DDP plays a marked role in the development of jejunum, the major site for digestion and absorption. Replacement of chicks diet with 15% DP had a higher weight percentage of small intestine parts such as jejunum when compared to control treatment group (14). There were reports that high fiber contents increased length and weight of intestine of broilers (34). The results of the present study agreed with similar findings of DP diet-fed broiler chicks, which showed increased weight and length of the intestine (11). Chemical analysis showed that DDP contains a substantial amount of fiber (19), and the fiber content promotes the absorption of nutrients by the intestinal segments, which elevates the length and weight of the intestine.

Morphometric results of the duodenum, jejunum, and ileum in broilers on day 42 of the experiment revealed that DDP treatment enhances the health of the digestive tract by altering the villus height and crypt depth. Absorption of nutrients is promoted by longer villi and shallower crypts with greater surface area, which activates maturation of the intestinal cells and digestive enzyme activity (35, 36). The crypt is the production site where divisions of stem cells occur to allow villus renewal. The higher duodenal and jejunal villi height of broilers fed a diet containing 10% DDP showed the enhancement of the villi surface area. This suggests that the addition of 10% DDP enhances the absorption of nutrients, improves the resistance to disease, and increases overall growth performance. The 10% DDP diet-fed broilers showed shallow crypt depth and increased villus crypt ratio in the duodenum, jejunum, and ileum, which indicates the positive role of DDP in the development of broilers GI tract. MOS has been observed to raise villus height and reduce crypt depth in poultry (37). Reports showed that MOS decreased the crypt depth in the small intestines of broilers and encouraged their rapid growth (38, 39). Previous studies have shown that broilers fed fructooligosaccharides, one of the common prebiotics had improved intestinal structure and morphology (40). In broilers fed MOS together with enzymes have shown an increase in perimeter and height of villi of the duodenum and ileum, which increased the absorption surface of the intestinal segments (41). An increased villus height and crypt depth were observed in broilers fed enzyme-treated palm kernel expeller (42), while in broilers fed fermented palm kernel expeller up to 36% decreased intestine villus height was observed (43). Previous research showed that high fiber content of palm kernel meal stimulated the development of the mucosa epithelial cells confirmed by higher and wider villus in broiler chickens fed 25% palm kernel meal diet (44). The present study also supports the above finding that fiber content of DDP plays an important role in the altered morphology of broilers intestine.

The digestive enzymes play an important role in the decomposition of absorbed nutrients into small molecules for utilization of broilers (45). Results showed that in broilers fed 10% DDP diet an enhanced activity of the pancreatic enzymes amylase and lipase were observed. Different factors, such as the animal age, secretion of digestive juice, and composition of diets, influence the activities of enzymes (46). Amylase, produced by the pancreas, hydrolyses starch into smaller units that can be absorbed in the bird intestine (47). The activity of digestive enzymes was increased in broilers fed MOS (48). Increased activity of amylase, lipase, and protease was observed in broiler chicks supplemented with fructose oligosaccharide up to 4.0 g/kg when compared to the control corn diet-fed broilers (40). The increase in α-amylase activity in the gut in the DDP-supplemented broilers indicates that DDP enhances the secretion of the enzyme from the pancreas. Pancreatic lipase and amylase stimulate the digestion and absorption of nutrients, which leads to a healthy intestine of broilers and enhances the growth performance of the birds.

## Conclusions

DDP had beneficial effects in broilers that can be used as a locally produced and affordable feed additive and an inexpensive feed ingredient. Inclusion of both 10% DDP and 0.2% MOS in broilers rations improved carcass characteristics and intestine development. The enhancement of digestive enzymes activity indicates that DDP provide a healthy gut for the digestion and absorption of ingested nutrients. It can be concluded that DDP can act as a prebiotic similar to MOS, and supplementation of DDP can be fed as an alternative promising growth promoter in poultry feeding.

## Data Availability Statement

The datasets generated for this study are available on request to the corresponding authors.

## Ethics Statement

The animal study was reviewed and approved by the scientific committee of the Department of Integrative Agriculture, College of Food and Agriculture, United Arab Emirates University.

## Author Contributions

All authors listed have made a substantial, direct and intellectual contribution to the work, and approved it for publication.

## Conflict of Interest

The authors declare that the research was conducted in the absence of any commercial or financial relationships that could be construed as a potential conflict of interest.

## Abbreviations

DP: date pits
DDP: degraded date pits
MOS: mannan-oligosaccharides
CSBD: corn-soy basal diet
ME: metabolizable energy
CP: crude protein
EE: ether extract
CF: crude fat
NFE: nitrogen-free extract (starch + sugar)
SAS: statistical analyses software
GLM: general linear model
SE: standard error
NSP: non-starch polysaccharide
SSD: solid-state degradation.
